# Spectroscopic Evidence of Energy Transfer in BODIPY-Incorporated Nano-Porphyrinic Metal-Organic Frameworks

**DOI:** 10.3390/nano10101925

**Published:** 2020-09-26

**Authors:** Changwon Seo, Miyeon Kim, Jubok Lee, Chang Yeon Lee, Jeongyong Kim

**Affiliations:** 1 Department of Energy Science, Sungkyunkwan University, Suwon 16419, Korea; cwseo323@skku.edu (C.S.); vopop@skku.edu (J.L.); 2 Department of Energy Chemical Engineering, Incheon National University, Incheon 22012, Korea; mykim@inu.ac.kr; 3 Innovation Center for Chemical Engineering, Incheon National University, Incheon 22012, Korea

**Keywords:** metal-organic frameworks (MOFs), scanning laser confocal microscopy, resonance energy transfer, photoluminescence mapping, time-resolved photoluminescence

## Abstract

Metal–organic frameworks (MOFs) represent a class of solid-state hybrid compounds consisting of multitopic organic struts and metal-based nodes that are interconnected by coordination bonds, and they are ideal for light harvesting due to their highly ordered structure. These structures can be constructed with chromophore organic ligands structures for the purpose of efficient light harvesting. Here, we prepared porphyrin-based nano-scaled MOFs (nPCN-222) with BODIPY and I_2_BODIPY photosensitizers by incorporating BODIPY/I_2_BODIPY into nPCN-222 (nPCN-BDP/nPCN-I_2_BDP) and demonstrated resonance energy transfer from the donor (BODIPY/I_2_BODIPY) to the acceptor (nPCN-222) resulting in greatly enhanced fluorescence of nPCN-222, as visually manifested by time-resolved and space-resolved fluorescence imaging of the nano-scaled MOFs.

## 1. Introduction

In nature, energy transfer occurs in antenna complexes of plants and antenna pigments such as chlorophyll. The antenna complex aids in the absorption of sunlight over a wide wavelength range and transfers this energy to a reaction center via resonance energy transfer [1]. The phenomenon of resonance energy transfer can been exploited in the generation of photo-induced products such as singlet-oxygen [2], in biosensors [3] and photodynamic therapy [4]. To achieve high resonance energy transfer efficiency, modulating the distance between the donor and acceptor [5], adjusting the bandgap of quantum dots by changing their size [6], achieving overlap between the energy of emission of the donor and the absorption of the acceptor [1] and constructing self-assembled donor-acceptor supramolecular systems [7] have been studied. Assembling ordered structures of chromophores, for example by structuring by self-assembly of covalent building blocks, can also improve the energy transfer efficiency [8].

Metal-organic frameworks (MOFs) are constructed with multidentate organic building blocks and metal-cluster secondary building units have received remarkable attention as efficient light harvesting structures because of their prominent benefits including high porosity, large surface area, and tailorable functionality [9]. These advantages of MOFs have led to vast potential applications in areas such as gas storage [10], catalysis [11,12], photodynamic therapy (PDT) [13,14] and light-harvesting [15,16]. MOFs provide ideal platforms for investigating light-harvesting properties. By exploiting the rigid structural properties of crystalline MOFs with fixed interchromophoric distance and orientation, systematic study of the correlation between the light-harvesting property and fine structural environment of chromophores is allowed [17,18,19]. Furthermore, benefitting from the highly developed organic synthesis methods and post-synthetic modification processes, various complementary chromophores can be conveniently incorporated into MOFs via direct [16,20,21] or post-synthesis procedures [19,22]. Indeed, representative complementary chromophores, i.e., boron-dipyrromethene (BODIPY) and porphyrin ligands, have been successfully included in frameworks by direct synthesis with BODIPY and the porphyrin ligand [16] or by post-synthesis modification of porphyrin-based MOFs with the BODIPY ligand [22].

Among the various MOFs, porphyrin-based MOFs epitomize the artificial light-harvesting system due to the similarity of their optical absorption and chemical reaction characteristics to those of chlorophylls. Considering the absorption bands of porphyrin-based MOFs [23], judicious selection and incorporation of antenna (energy donor) chromophoric ligands having a complementary absorption to porphyrin (energy acceptor) can expand the solar spectrum coverage of MOFs. Moreover, energy transfer from the antenna ligand to the porphyrin results in improved light-harvesting of porphyrin-based MOFs. We previously demonstrated the aforementioned concepts by incorporating complementary chromophores such as pyrene or BODIPY derivatives into porphyrin-based MOFs by the mixed-ligand approach or post-synthesis modification [21,22]. In both cases, the enhanced light-harvesting properties of the porphyrin-based MOFs induced superior performance for photocatalysis or photodynamic therapy via the generation of photo-induced reactive oxygen species. A representative porphyrinic MOF, PCN-222, was down-sized to the nanometer scale in order to explore its photocatalytic [24,25] and PDT utility [22,26,27]. Nano-sized PCN-222 (nPCN-222) not only provided highly exposed active sites with a high surface to volume ratio for photocatalytic applications, but also exhibited efficient cellular uptake in PDT application.

In this study, we incorporate BODIPY or iodinated BODIPY (I_2_BODIPY) into the ordered pores of nano-scaled porphyrin-based MOFs (nPCN-222) by a solvent-assisted ligand incorporation (SALI) process and visualize the energy transfer process by employing space- and time-resolved confocal photoluminescence microscopy. BODIPY is well known for its high quantum yield [28] and strong absorption band centered in the green region where porphyrin absorbs only marginally [29]. The population of photoexcited singlet states and triplet states of BODIPY can be modulated through iodination of BODIPY (I_2_BODIPY) [30], affording a variety of engineering possibilities for energy transfer routes in nano-scaled MOFs. Although various applications of nano-sized PCN-222 have been developed, the fate of the excited states of nPCN-222, including energy transfer between BODIPY and nPCN-222, have not been investigated so far. Herein, the BODIPY and I_2_BODIPY incorporated nano-scaled MOFs are denoted as nPCN-BDP and nPCN-I_2_BDP, respectively. It is firstly demonstrated that energy transfer from BODIPY to porphyrin ligands greatly enhances porphyrin emission of nPCN-222.

## 2. Materials and Methods

### 2.1. Synthesis

**Scheme 1:**
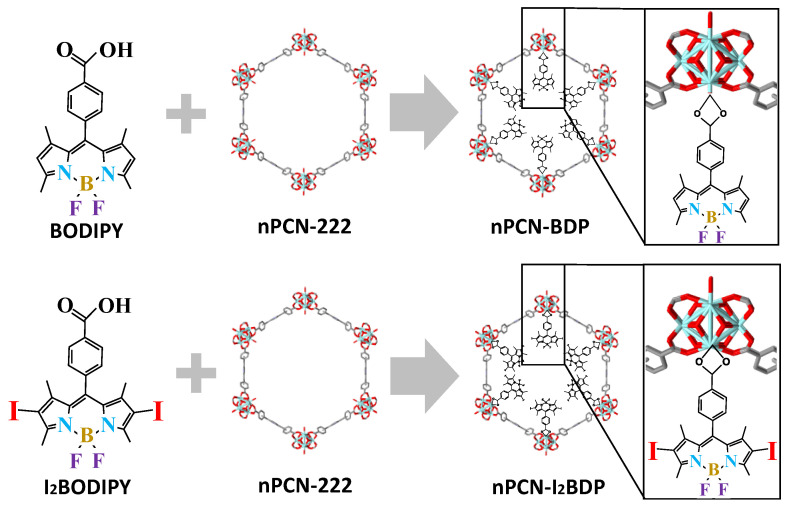
Schematic of the synthesis of nPCN-BDP and nPCN-I_2_BDP by incorporating BODIPY or I_2_BODIPY into nPCN-222 MOFs, respectively.

Nano-scaled PCN-222 was synthesized by a previously reported method [31]. 4,4-Difluoro-8-(4’-carboxyphenyl)-1,3,5,7-tetramethyl-4-bora-3a,4a,-diaza-s-indacene (BODIPY) [32] and 4,4-difluoro-8-(4’-carboxyphenyl)-2,6-diiodo-1,3,5,7-tetramethyl-4-bora-3a,4a,-diaza-s-indacene (I_2_BODIPY) [33] were synthesized according to a literature procedure. For the synthesis of nPCN-BDP and nPCN-I_2_BPD, activated nPCN-222 (45 mg, 0.0188 mmol) was placed into a 5 mL vial with 3 mL of a solution comprising BODIPY or diiodo-BODIPY (0.0564 mmol) in acetonitrile/DMSO (3:2) (Scheme 1). The mixture was sealed and heated in a 60 °C oven for 24 h. The solid was isolated by centrifugation with acetonitrile (>99.5%, TCI, Tokyo, Japan)/methanol (99.5%, Daejung, Gyeonggi-do, Korea) (1:1 volume ratio) and acetone several times in order to remove unreacted BODIPY or I_2_BODIPY; the remaining acetone (99.5%, Daejung, Gyeonggi-do, Korea) was removed under vacuum at 80 °C. 

### 2.2. Optical Measurements

Macro absorption and fluorescence spectra were acquired with a UV-VIS spectrometer (V-670, JASCO International Co., Ltd., Tokyo, Japan) and a Cary Eclipse Fluorescence Spectrophotometer (Agilent Technologies, St. Clara, CA, USA), respectively. Powdered samples of nPCN-222, BODIPY, I_2_BODIPY, nPCN-BDP (BODIPY incorporated into nPCN-222), and nPCN-I_2_BDP (I_2_BODIPY incorporated into nPCN-222) were dispersed in acetone solution, and 10 mg of each powder sample was dissolved in 10 mL of acetone and then sonicated for 24 h before loading into quartz cuvettes for acquisition of the absorption and fluorescence spectra. For confocal fluorescence and fluorescence lifetime imaging, 1 mL solutions of nPCN-222, BODIPY, I_2_BODIPY, nPCN-BDP, and nPCN-I_2_BDP were drop-casted on a glass substrate and dried under ambient conditions for 30 min. A commercial confocal microscope (Alpha-300S, Witec GmbH, Ulm, Germany) with a 488 nm diode laser (BDL-488, Becker-Hickl GmbH, Berlin, Germany) as a continuous-wave excitation source and a 633 nm He-Ne laser (LGK 7665-20, Lasos Lasertechnik GmbH, Jena, Germany) were used. The laser light was focused with a 0.9 numerical aperture (NA) objective lens and the fluorescence was collected by the same objective lens, focused on an optical fiber with a 100 µm core diameter, which acted as a confocal detection pinhole, and then guided to a spectrometer equipped with a cooled charge-coupled device (CCD) (PIXIS 100B, Trenton, NJ, USA). Fluorescence lifetime curves and images were obtained by using the same optical microscopy system combined with a commercial time-correlated single photon counting (TCSPC) system (Simple-Tau 150, Becker-Hickl GmbH, Berlin, Germany), 488 nm diode laser operated in the pulsed mode with a repetition rate of 80 MHz, and high-speed photomultiplier tube detector (HPM-100, Becker-Hickl GmbH, Berlin, Germany). The TCSPC system was electronically synchronized with the scanning stage of the confocal microscope(Alpha-300S, Witec GmbH, Ulm, Germany), and an emission decay profile was obtained at every pixel position, and a map of the lifetime was obtained with 64 × 64 pixels by using SPCImage software (Ver. 8.1, Becker-Hickl GmbH, Berlin, Germany) [21].

## 3. Results

**Figure 1:**
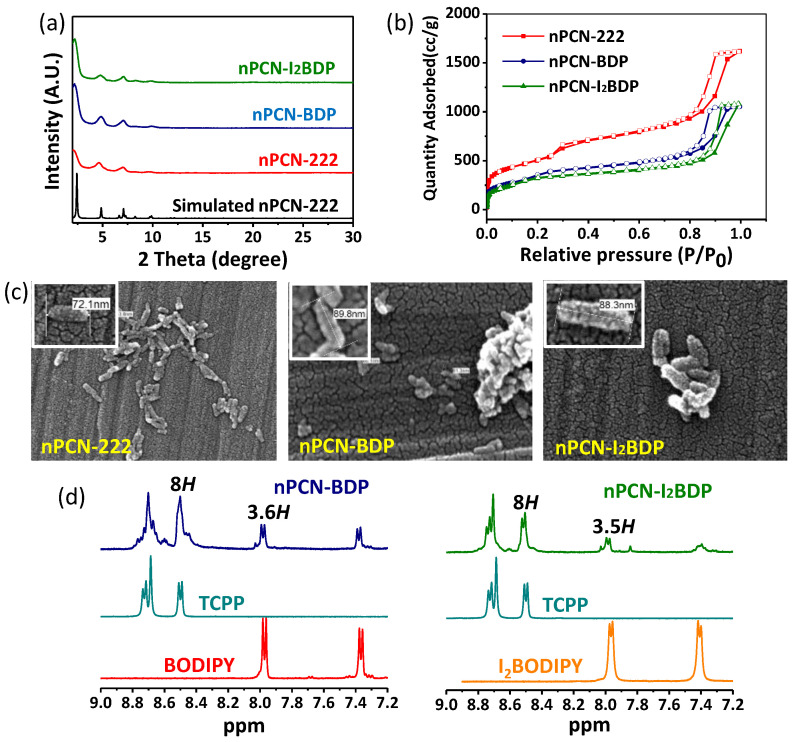
(**a**) PXRD patterns of nPCN-222, nPCN-BDP, and nPCN-I_2_BDP. (**b**) Surface area of nPCN-222, nPCN-BDP, and nPCN-I_2_BDP was determined from N_2_ absorption-desorption isotherms. (**c**) SEM images of nPCN-222, nPCN-BDP, and nPCN-I_2_BDP; inset shows the size of each MOF, which was consistently below 100 nm. (**d**) NMR data for nPCN-BDP and nPCN-I_2_BDP.

nPCN-BDP and nPCN-I_2_BDP were synthesized by solvent-assisted ligand incorporation (SALI) [22] as schematically depicted in Scheme 1, where BODIPY/I_2_BODIPY ligands were incorporated into the pores of nPCN-222 and attached to the metal nodes of nPCN-222. The powder X-ray diffraction (PXRD) patterns of nPCN-BDP and nPCN-I_2_BDP are consistent with those of nPCN-222 and the simulated patterns, indicating no perturbation of the crystallinity of the frameworks after the SALI process (Figure 1a). The broadening of the PXRD patterns is derived from size reduction of the MOFs crystals. N_2_ adsorption-desorption isotherms were acquired to evaluate the porous properties of the MOFs.

The isotherms of all MOFs reveal type IV features, indicating the combined micro- and mesoporous nature. The surface areas of nPCN-BDP (1118 m^2^·g^−1^) and nPCN-I_2_BDP (1015 m^2^·g^−1^) decreased compared to that of the parent nPCN-222 (1755 m^2^·g^−1^), suggesting incorporation of the BODIPY ligand into nPCN-222 (Figure 1b). The morphology and size of the MOFs crystals after post-modification were investigated by scanning electron microscopy (SEM) (Figure 1c). The average sizes of all MOFs were below 100 nm. Nuclear magnetic resonance spectroscopy (NMR) was used to determine the amount of incorporated BODIPY by decomposing the MOFs in a D_2_SO_4_ (10%)/DMSO-d_6_ mixture. Resonance signals originating from the porphyrin, BODIPY, and I_2_BODIPY ligands were observed in the NMR spectra of nPCN-BDP and nPCN-I_2_BDP. By comparing the relative integrated areas of the two signals corresponding to porphyrin and BODIPY or I_2_BODIPY, it was determined that 3.6 BODIPY and 3.5 I_2_BODIPY ligands per Zr_6_ node were incorporated after the SALI process (Figure 1d). Given that the maximum possible number is four ligands per Zr_6_ node, the BODIPY and I_2_BODIPY ligands occupy most of the nodes in nPCN-BDP and nPCN-I_2_BDP.

**Figure 2:**
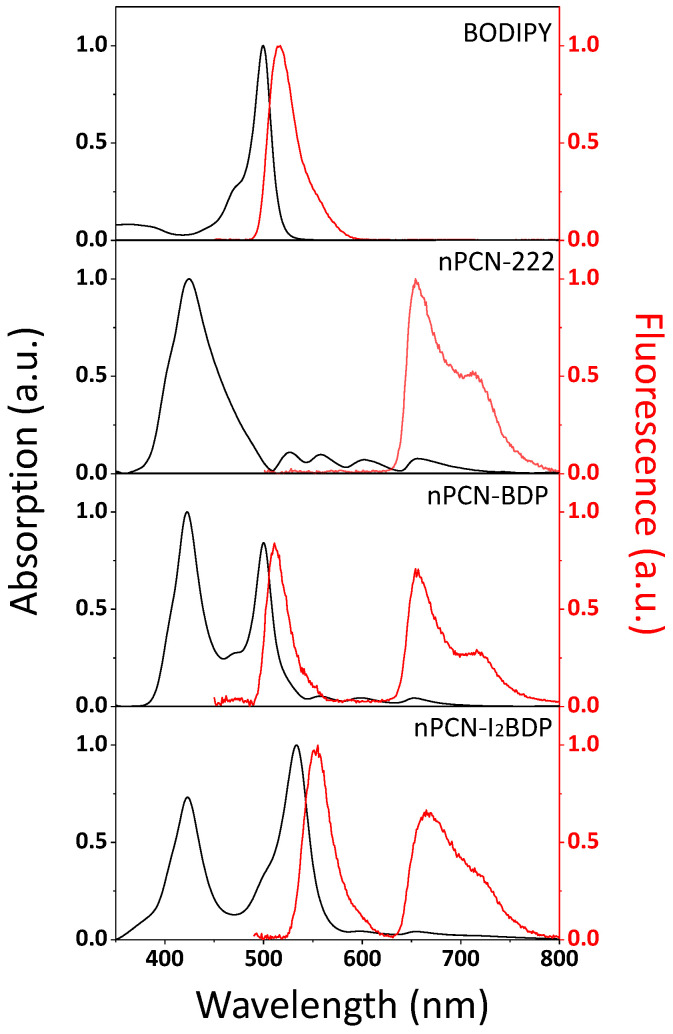
Absorption and fluorescence emission spectra of BODIPY, nPCN-222, nPCN-BDP and nPCN-I_2_BDP dispersed in acetone.

Figure 2 shows the absorption and fluorescence spectra of BODIPY, nPCN-222, nPCN-BDP, and nPCN-I_2_BDP dispersed in acetone. The absorption and fluorescence peaks of BODIPY were observed at 500 nm and 515 nm, respectively, which is consistent with the characteristic fundamental transition of BODIPY [34]. Absorption and fluorescence spectra of nPCN-222 showed characteristics Soret (425 nm) and Q (500 nm~700 nm) absorption bands and fluorescence peaks at 655 nm and 715 nm of the porphyrin which are the base ligands (tetrakis(4-carboxyphenyl)porphyrin(TCPP)) of nPCN-222 [35,36,37]. nPCN-BDP displayed the absorption (fluorescence) features of nPCN-222 and BODIPY at 425 nm (655 nm and 715 nm) and at 500 nm (515 nm), respectively. Likewise, the absorption (fluorescence) spectrum of nPCN-I_2_BDP shows the spectral features of nPCN-222 and I_2_BODIPY, with peaks at 425 nm (666 nm and 722 nm) and at 533 nm (550 nm), respectively. The clear observation of the absorption and fluorescence features of BODIPY and I_2_BODIPY in the spectra of nPCN-BDP and nPCN-I_2_BDP confirmed the presence of the BODIPY/I_2_BODIPY ligands in nPCN-222.

**Figure 3:**
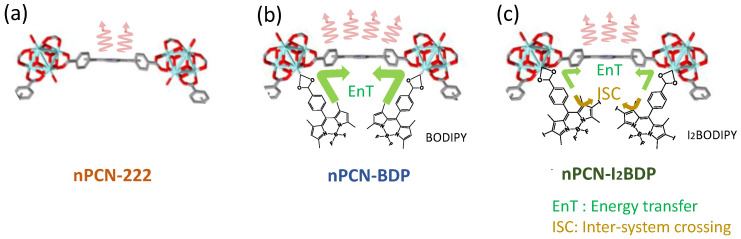
Expectation of transition pathways of the incorporated MOFs system. (**a**) Fluorescence emission of nPCN-222. (**b**) Enhanced fluorescence emission of nPCN-222 (acceptor) induced by resonance energy transfer from BODIPY (donor) in nPCN-BDP. (**c**) Energy transfer and inter-system crossing can occur in nPCN-I_2_BDP.

Figure 3 presents a schematic of the energy and inter-system crossing pathways in nPCN-BDP and nPCN-I_2_BDP. In nPCN-222, only the porphyrin ligands are photoexcited and emit fluorescence, as shown in Figure 3a, whereas in the nPCN-BDP MOFs, the porphyrin and BODIPY ligands can both be photoexcited and a part of the excitation energy in the BODIPY ligands can be transferred to the porphyrin ligands because of the spatial proximity and spectral overlap between BODIPY and porphyrin. Notably, there is spectral overlap between the emission energy of BODIPY and the absorption energy of the porphyrin, as shown in Figure 2. At the onset of energy transfer, fluorescence from the porphyrin (acceptor) would be enhanced due to energy transfer from BODIPY (donor), as illustrated in Figure 3b. In the nPCN-I_2_BDP systems, energy transfer from I_2_BODIPY to the porphyrin ligands is expected, and enhancement of the porphyrin emission will be less efficient than in nPCN-BDP because some of the photoexcitation energy of I_2_BODIPY can decay through intramolecular inter-system crossing due to the iodine atoms in the nPCN-I_2_BDP systems [22], as illustrated in Figure 3c.

**Figure 4:**
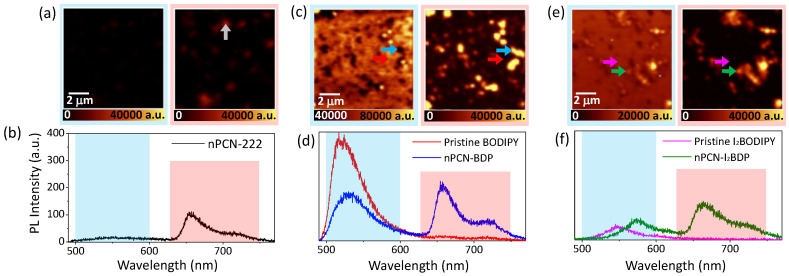
Fluorescence maps and representative local fluorescence spectra of (**a,b**) nPCN-222, (**c,d**) nPCN-BDP, and (**e,f**) nPCN-I_2_BDP samples. The fluorescence maps were obtained by integrating the donor (BODIPY/I_2_BODIPY) emission range of 500–600 nm (blue box) and acceptor (nPCN-222) emission range of 630–750 nm (red box). In each map, local representative fluorescence spectra were obtained from the location marked by the arrows.

The confocal fluorescence maps and representative spectra of nPCN-222, nPCN-BDP, and nPCN-I_2_BDP are presented in Figure 4. The blue and red background boxes in the fluorescence spectra and maps represent the wavelength range of the donor emission (500–600 nm) and acceptor emission (630–750 nm), respectively. Figure 4a presents the fluorescence image of nPCN-222. Due to the absence of BODIPY emission in nPCN-222, almost no fluorescence was observed below 600 nm. However, in the acceptor emission range, nanoscale grains of nPCN-222 were observed in the fluorescence image, as shown in the right panel of Figure 4a. A representative fluorescence spectrum obtained at the position marked by the arrow (white) is shown in Figure 4b, which displays characteristic emission peaks at 660 nm and 720 nm. Figure 4c displays the fluorescence images of nPCN-BDP in the donor emission range (blue panel) and acceptor emission range (red panel). When BODIPY was incorporated into nPCN-222 to form nPCN-BDP, fluorescence was detected in the donor emission range, as shown in Figure 4c (left panel). The donor emission was observed not only on the MOFs grains, but also in the background region of the substrate, which is the result of remnants of BODIPY, where a small trace of the BODIPY ligand was present due to incomplete removal of the free ligand after the SALI process. Here, we note that the fluorescence of BODIPY on the background substrate was more intense than that on the MOFs grains; the representative fluorescence spectra from the MOFs grains and the background are shown in Figure 4d. Considering that donor emission from the background substrate represents the emission of BODIPY in the monomer state, i.e., BODIPY that was not incorporated into nPCN-222, the observed quenching of BODIPY emission on the MOFs grains is a strong indication of energy transfer in nPCN-BDP, where the excitation energy of BODIPY is transferred to the porphyrin ligands. Compared with nPCN-222, the acceptor emission of nPCN-BDP was clearly enhanced, which provides further evidence of energy transfer between BODIPY and the porphyrin ligands in nPCN-BDP. Figure 4e shows the fluorescence images of nPCN-I_2_BDP. The left and right images of Figure 4e represent the donor and acceptor emission ranges of nPCN-I_2_BDP, respectively. The MOFs grains of nPCN-I_2_BDP exhibit strong fluorescence in the 630‒750 nm wavelength range (right panel). Here, similar to nPCN-BDP, donor emission from the remnant I_2_BODIPY on the background substrate was observed and the enhancement in emission intensity of the porphyrin on the nPCN-I_2_BDP MOFs grains somewhat weaker than that in nPCN-BDP, as seen in the representative fluorescence spectra acquired from the locations of the nPCN-I_2_BDP grains (indicated by green arrows in Figure 4e), as shown in Figure 4f.

The reduced enhancement of the acceptor emission in nPCN-I_2_BDP compared to nPCN-BDP is believed to be due to the presence of additional pathways for non-radiative decay of I_2_BODIPY emission through inter-system crossing via the heavy-atom effect. Interestingly, unlike nPCN-BDP, the donor emission of nPCN-I_2_BDP was slightly stronger on the MOFs grains than on background substrate, as shown in the fluorescence spectra obtained from two selected locations in Figure 4e,f indicating that energy transfer from I_2_BODIPY to the porphyrin ligand in PCN-I_2_BDP is not as efficient as in nPCN-BDP.

**Figure 5:**
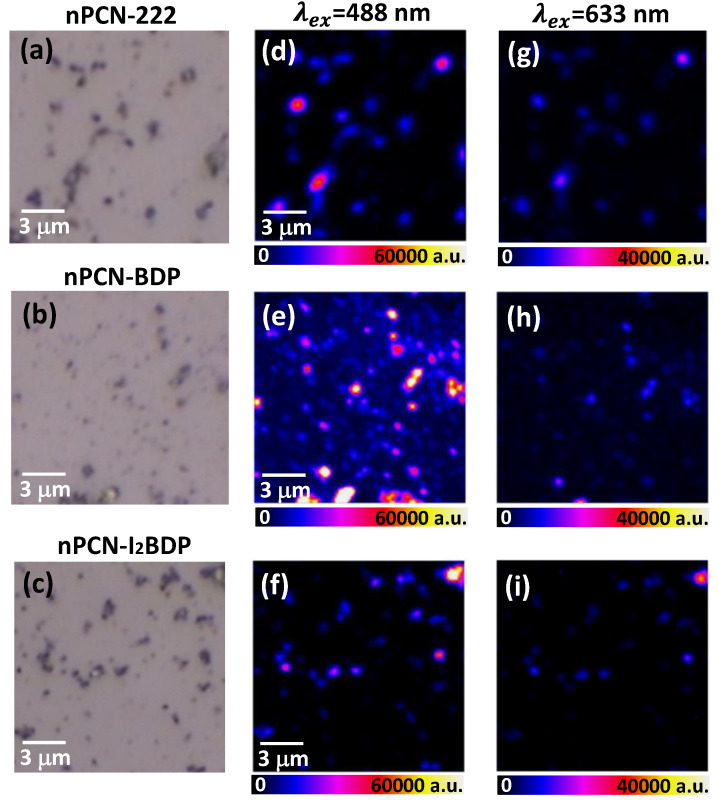
Microscopy images of (**a**) nPCN-222, (**b**) nPCN-BDP, and (**c**) nPCN-I_2_BDP. Fluorescence images of porphyrin ligand of (**d**) nPCN-222, (**e**) nPCN-BDP, and (**f**) nPCN-I_2_BDP after 488 nm excitation. Fluorescence images of (**g**) nPCN-222, (**h**) nPCN-BDP, and (**i**) nPCN-I_2_BDP after 633 nm excitation. All images were obtained by integrating 630–750 nm emission range.

The remarkable effect of energy transfer on the fluorescence intensity of nPCN-BDP was demonstrated by laser excitation at two different wavelengths. For excitation at 633 nm, the photon energy is not high enough to excite BODIPY but will only excite the porphyrin ligand. Under 488 nm excitation, both BODIPY and the porphyrin ligand of nPCN-BDP are excited, leading to resonance energy transfer from BODIPY to the porphyrin ligand in nPCN-BDP. Figure 5 shows the optical images (5a, 5b, 5c) and fluorescence images obtained under excitation of the dispersed MOFs grains of nPCN-222, nPCN-BDP, and nPCN-I_2_BDP at 488 nm (5d, 5e, 5f) and 633 nm (5g, 5h, 5i). The fluorescence intensity in the range of 630‒750 nm was integrated to represent the porphyrin ligand emission. For nPCN-222, the fluorescence intensity was significantly enhanced under 488 nm excitation compared to that achieved with 633 nm excitation, which is attributed to the higher absorption of the Soret band, which exceeds the absorption by the Q band of the porphyrin ligand (see Figure 2). For nPCN-BDP, the fluorescence of the MOFs grains was much more intense under 488 nm excitation than 633 nm excitation, where the former was about seven times higher. This cannot be simply explained by greater absorption of the 488 nm excitation than the 633 nm excitation, but is attributed to energy transfer from photoexcited BODIPY to the porphyrin ligands. Similar experiments were performed with nPCN-I_2_BDP MOFs (Figure 5f,i), where only a moderate enhancement of the fluorescence was observed with 488 nm excitation, which again suggests that energy transfer in nPCN-I_2_BDP is less effective than that in nPCN-BDP.

**Figure 6:**
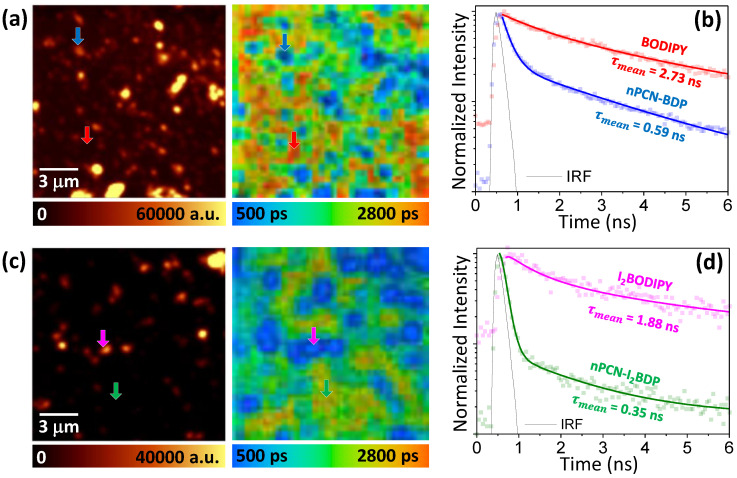
(**a**) Fluorescence intensity and lifetime maps of nPCN-BDP. (**b**) Representative fluorescence decay curves of donor emission of BODIPY monomer (red curve) and nPCN-BDP (blue curve). (**c**) Fluorescence intensity and lifetime maps of nPCN-I_2_BDP. (**d**) Representative decay curves of donor emission of I_2_BODIPY monomer (magenta curve) and nPCN-I_2_BDP (green curve). Instrument response function (IRF) is indicated by black line. Fluorescence lifetime maps were obtained from donor emission portion of nPCN-BDP (500–560 nm) and nPCN-I_2_BDP (530–590 nm).

Energy transfer in the MOFs systems was manifested in fluorescence lifetime mapping [21]. The fluorescence lifetime maps and the representative fluorescence decay curves of the donor (BODIPY of nPCN-BDP or I_2_BODIPY of nPCN-I_2_BDP) emissions were obtained. Figure 6a presents the spatial map of the mean fluorescence lifetime of BODIPY emission for nPCN-BDP. The fluorescence intensity maps (shown in Figure 5e) are also presented to aid identification of the location of the MOFs grains. The fluorescence lifetime map presents a convincing spatial correlation, indicating that the fluorescence lifetime is longer at the background BODIPY monomers than the nPCN-BDP MOFs grains. The two representative fluorescence lifetime curves shown in Figure 6b were obtained from the nPCN-BDP MOFs grain and the background monomer BODIPY region (indicated by arrows in Figure 6a). Notably, the fluorescence decay of the background BODIPY monomer shows a almost linear response, which is typical for non-interacting molecules free from energy or electron transfer [38], whereas at the nPCN-BDP MOFs grains, the fluorescence lifetime was reduced, which suggests the onset of energy transfer from BODIPY to the porphyrin ligand. Figure 6c shows the fluorescence lifetime map along with the fluorescence intensity image (Figure 5f), and Figure 6d shows the two representative fluorescence lifetime curves obtained from the nPCN-I_2_BDP grain and background monomer I_2_BODIPY region. The fluorescence lifetime of the I_2_BODIPY monomer is intrinsically shorter than that of the BODIPY monomer due to inter-system crossing induced by the iodine atoms in the former.

**Figure 7:**
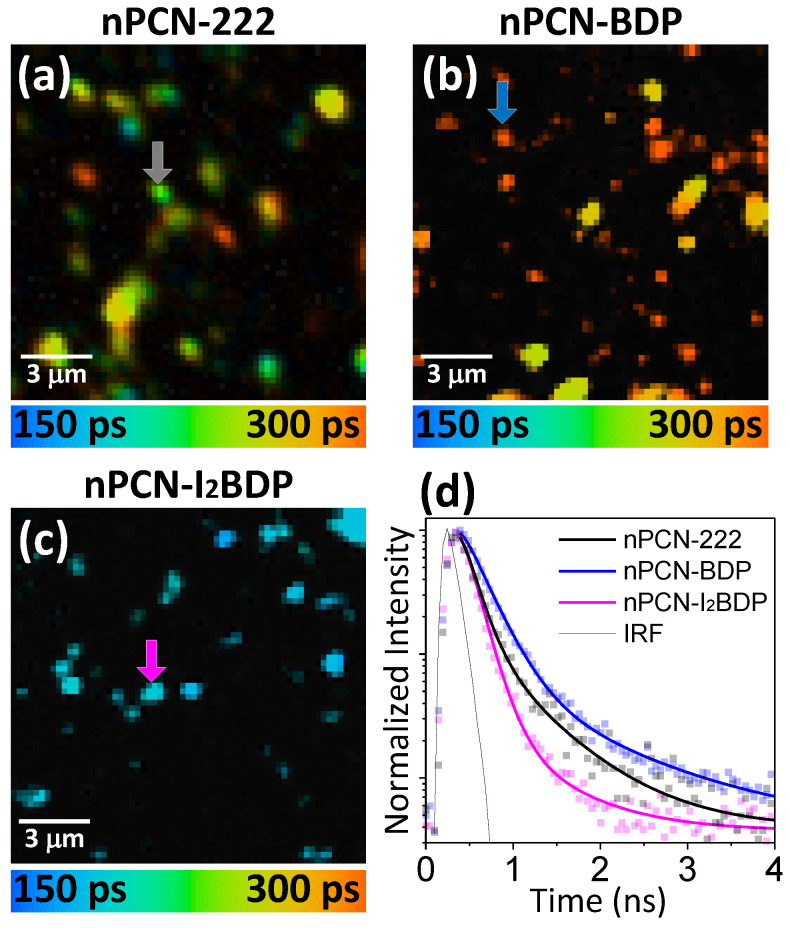
(**a**) Fluorescence lifetime map of pristine nPCN-222 emission (630–750 nm) (same sample with Figure 5a. (**b**) Fluorescence lifetime map of acceptor emission of nPCN-BDP. (**c**) Fluorescence lifetime map of acceptor emission for nPCN-I_2_BDP. The spatial position of (**b**,**c**) is same with Figure 6a,c, respectively. (**d**) Representative fluorescence lifetime decay curves of acceptor emission obtained at the locations indicated by arrows in (**a**–**c**) that represent pristine nPCN-222 (Black), acceptor emission for nPCN-BDP (blue) and for nPCN-I_2_BDP (magenta).

Energy transfer in nPCN-BDP system was also evinced by modified fluorescence lifetime of the acceptor. Figure 7 presents the spatial map of the mean fluorescence lifetime of nPCN-222, nPCN- BDP and nPCN-I_2_BDP at acceptor emission between 630 nm and 750 nm and representative time decay curves of fluorescence.

As shown in fluorescence lifetime map of nPCN-222 in Figure 7a, the mean fluorescence lifetime of pristine nPCN-222 MOFs ranges between 220–250 ps. On the other hand, the lifetime map of nPCN-BDP shown in Figure 7b displays a distinctly longer fluorescence lifetime than pristine nPCN-222 MOFs grains, which strongly indicates the energy transfer from BODIPY to nPCN-222 ligands. It is known that when the energy transfer occurs in donor-acceptor system, acceptor fluorescence lifetime tends to increase along with the rise time [21,39,40]. However, the lifetime map of acceptor emission of nPCN-I_2_BDP MOFs shown in Figure 7c indicates shorter fluorescence lifetime than that of pristine nPCN-222. Representative time decay curves of fluorescence are displayed in Figure 7d which are obtained from arrow marked MOFs grains.

The obtained fluorescence decay curves were fitted to a bi-exponential function, expressed as:

(1) $$F\left(t\right)=A_{1}e^{-t/\tau_{1}}+A_{2}e^{-t/\tau_{2}}$$

Here, *A*_1_ and *A*_2_ are the amplitudes, and $\tau_{1}$ and $\tau_{2}$ are fast and slow fluorescence lifetime components, respectively. The fast and slow fluorescence lifetime components emerged due to non-radiative recombination via charge or energy transfer, and radiative recombination via fluorescence emission, respectively [41]. The mean fluorescence lifetime, $\tau_{mean}$ was calculated as:

(2) $$\tau_{mean}=\frac{A_{1}\tau_{1}+A_{1}\tau_{1}}{A_{1}+A_{2}}$$

The obtained fluorescence lifetime components of donor emission are summarized in Table 1. The reduction of mean fluorescence lifetime from 2.74 ns to 0.59 ns and the notable emergence of the fast decay component in nPCN-BDP compared to the monomer state of BODIPY indicate the energy transfer from BODIPY to the porphyrin ligands in nPCN-BDP. The mean lifetime and fast decay time of I_2_BODIPY emission are distinctly shorter than that of BODIPY, which we attribute to electron transfer via inter-system crossing. The lifetime of I_2_BODIPY is even further reduced in nPCN-I_2_BDP, which suggests the non-radiative recombination perhaps due to the combined effects of the energy transfer and electron transfer in nPCN-I_2_BDP.

Fluorescence lifetime components of acceptor emission are summarized in Table 2. The mean fluorescence lifetime of nPNC-222 was increased from 0.23 ns to 0.29 ns in nPCN-BDP due to energy transfer from BODIPY. However, the fluorescence lifetime was reduced in nPCN-I_2_BDP implying that the energy transfer is not efficiently occurring in I_2_BODIPY due to the presence of triplet state of I_2_BODIPY [42].

## 4. Conclusions

We have demonstrated energy transfer in porphyrin-based nano-scaled MOFs, incorporating BODIPY and iodine-substituted BODIPY. By employing time- and space-resolved laser confocal microscopy imaging, a significant enhancement of the emission intensity and the lifetime of nPCN-222 and a reduction of the emission lifetime of BODIPY in the nPCN-BDP MOFs system were observed, providing unambiguous evidence of energy transfer between the ligands of nPCN-222. Energy transfer in the nPCN-I_2_BDP MOFs was somewhat less efficient due to intra-channel intersystem crossing in I_2_BODIPY, suggesting fine engineering of the energy transfer process in multi-ligand nano-scaled MOFs, which could find the practical application in photocatalysis and photodynamic therapy benefiting from high efficiency of energy transfer and nanoscale sizes.

## Figures and Tables

**Table 1:** Fitting parameters for fluorescence decay curves of donor emission of BODIPY, nPCN-BDP, I_2_BODIPY, and nPCN-I_2_BDP shown in Figure 6b,d.

Components	BODIPY	nPCN-BDP	I_2_BODIPY	nPCN-I_2_BDP
*A*_1_ (%)	33.2	82.9	53.7	90.6
$\tau_{1}$ (ns)	0.84	0.20	0.62	0.13
*A*_2_ (%)	66.8	17.1	46.3	9.4
$\tau_{2}$ (ns)	3.67	2.50	3.34	2.48
$\tau_{mean}\;$(ns)	2.73	0.59	1.88	0.35

**Table 2:** Fitting parameters for fluorescence decay curves of acceptor emission of nPCN-BDP, I_2_BODIPY, and nPCN-I_2_BDP shown in Figure 7d.

Components	nPCN-222	nPCN-BDP	nPCN-I_2_BDP
*A*_1_ (%)	91	95.6	91.4
$\tau_{1}$ (ns)	0.17	0.25	0.14
*A*_2_ (%)	9	4.4	8.6
$\tau_{2}$ (ns)	0.84	1.12	0.45
$\tau_{mean}\;$(ns)	0.23	0.29	0.16
